# Why psychiatry needs ethnography

**DOI:** 10.1192/bjp.2025.10318

**Published:** 2025-10

**Authors:** Lisa Dikomitis, Sukhwinder Singh Shergill

**Affiliations:** Warwick Medical School, University of Warwick, Coventry, UK; Kent and Medway Medical School, University of Kent and Canterbury Christ Church University, Canterbury, UK; Institute of Psychiatry, Psychology and Neuroscience, King’s College London, London, UK; Kent and Medway NHS and Social Care Partnership Trust, Maidstone, UK

**Keywords:** Anthropology, intersectionality, Sikhs, interdisciplinary research, participant observation

## Abstract

Psychiatrists and anthropologists both rely on observation, discourse analysis and access to participants’ internal and external worlds. Ethnographic fieldwork, a key method in medical anthropology, offers a powerful tool to establish a robust evidence base of how to address mental health inequalities in ethnic minority communities.

Psychiatry makes the strange familiar and the familiar strange, providing understanding of perceptual and cognitive experiences that may appear, at face value, to be extraordinary. Anthropology makes the familiar strange and the strange familiar.^1^ Acquiring new knowledge of different cultures is the core business of the medical anthropologist. The objective of such anthropological research is to describe the lives of people with a level of intimacy and sensitivity that can only be achieved through long-term, sustained engagement with members of the community under study. Anthropologists conduct ethnographic fieldwork: they live in the community and are immersed in the daily lives of the people they study. This method, participant observation, allows ethnographers to gain first-hand experiences and collect a wide range of perspectives on understandings and behaviours around health and illness.^2^ Such data cannot be collected through other well-established methodological approaches in mental health research, including clinical trials, surveys, experimental and epidemiological studies. The key aspect of psychiatric practice is to make sense of unusual perceptions, beliefs and psychophysiological processes underlying different understandings of the world around us. Increasingly, a multidisciplinary approach to mental health care often entails an integration of perspectives from different professionals. This commonly takes the form of observations of appearance and behaviours as well as interviews on mental health history and mental status examinations.

There are noticeable similarities between the practice of psychiatry and the practice of ethnography, particularly in how anthropologists and psychiatrists try to place the experience of a person within their own history and the culture they come from. Both attempt to understand how developmental factors have formed a person, how family, as well as the wider community, might influence a person. Another key similarity is the reliance on observation and analysis of discourse; they rely heavily on access to participants’ worlds, both internal and external. Thus, the quality of data are predicated on trust and on the extent to which people are willing to share experiences.^3^ Indeed, in anthropology and in psychiatry we probe very personal issues, necessitating a profound level of engagement and trust. In psychiatry, the lens is therapeutic; people come to the psychiatrist with an intention to share some of their experiences to benefit their health. By contrast, in anthropology, the ethnographer goes to the community to engage with people through living in their community. This requires time, and such reciprocity of trust must be earned. Consequently, meaningful ethnographic work often requires a year or longer of conducting participant observation in a community to enable adequate levels of trust and engagement. Only in doing so, can the ethnographer gain well-rounded, intersectional perspectives.

There is an increasing awareness of the value of taking an intersectional approach to better understand how multiple layers of social identities (for instance, gender, class, race, ability) converge in mental illness. Such intersectional research is critical to provide a nuanced understanding to identify inequalities that are often observed within and between groups with regards to mental health outcomes. The accumulation of disadvantages and poorer mental health outcomes of a specific population group needs to be researched through an intersectional framework which allows researchers to capture such complexity. In trying to formulate mental health assessment and management through an intersectional lens, we argue here that *psychiatry needs ethnography*. This is particularly relevant to the understanding of mental health of individuals from underserved communities – for example, some ethnic minority communities with perpetuating and enduring health inequalities.

However, using one such ethnic minority community as an example, and as we have previously discussed,^4^ the aggregation of distinct ethnic communities into wide-ranging categories, subsequently considered as homogenous groups, has actually prevented the understanding of critical factors contributing to mental health inequalities. This propagates the invisibility of large heterogenous ethnic subgroups in the UK, with serious consequences for developing mental health interventions which can adequately address these factors in specific ethnic communities. Here, we take the example of the mental health of Sikhs in the UK, a group that is often buried in the collective terms ‘South Asian’ and ‘Indian’. This is problematic as it does not consider the specific milieu (in terms of racism, sexism, religion and contextual disadvantages) of important groups within the large minority communities brought together in such collective terms. Most Sikhs in the UK have their origins in the Punjab, in Northwest India, or in East Africa. The first group of Sikhs migrated to the UK in the 1950s and 1960s. The 2021 UK census recorded 524 000 Sikhs, which is an increase of 24% since the 2011 census. Sikhs now constitute about 1% of the entire UK population. The county of Kent, in southeastern England, is home to a large Sikh community of over 74 000 Sikhs. Our study in Kent, in which we integrated psychiatry with anthropology, provides an excellent example of the need for interdisciplinary research underpinned by ethnography.

Racism has been identified as a key factor that negatively affects mental health outcomes in ethnic minority communities in the UK, such as Sikhs. Deeply rooted in societal structures, racism causes avoidable and unfair inequalities in power, resources and opportunities between racial or ethnic groups. Racism in mental health care manifests itself in many forms and behaviours through beliefs, stereotypes, prejudices and discrimination. The experience of racism can act as a stressor and cause significant psychological suffering. However, the attention given to racism’s role in mental health outcomes is significantly neglected, commonly rendering it invisible within mental health services and policies. The cumulative impact of intersectional disadvantages on mental health outcomes is complex and can be seen at individual, community and institutional levels. Sikhs experiencing the negative effects of racism are also often impacted by other forms of discrimination and oppression, related to their gender, socioeconomic circumstances, gender and biography of migration. Intersectionality theory provides a framework to examine multiple axes of differences and disadvantages that people experience. Such intersectional research is critical to provide a nuanced understanding of health seeking, access to mental health services and resources, and mental health outcomes. Through our Sikh mental health research, we gained an in-depth understanding of the lived experiences of generations of Sikh migrants in the UK.^4^

How does ethnography proceed? The first thing is that there is no magic formula and certainly no standard operating procedure for conducting successful ethnographic fieldwork. It is radically different from any other methodological approach used in mental health research. The data collection starts when the ethnographer enters the ‘field’, lives in the community and is immersed in the everyday reality of the people studied. The rules of academia and other methodological approaches are not necessarily those of that reality. The solution always constitutes unilateral adaptation, flexibility and creativity by the anthropologist. After all, the ethnographer is usually an uninvited guest. For our study, we lived in a large Kent Sikh community for several years; we spent time with Sikhs in their houses, in their place of worship (Gurudwara) and in a range of localities significant to the Sikh community. Ethnographers keep a field diary to document their ideas, reflections and observations. Other data include informal conversations, recorded and transcribed interviews, diaries, photographs, artefacts, public records, books and reports. Our ethnographic approach incorporates community members as co-researchers. It is paramount that research is not for Sikhs, or on Sikhs, but *with* Sikhs, ensuring that the research addresses the needs of the Sikh community. This commitment to working with Sikhs also included efforts to increase our understanding of these Sikhs’ migration background. Therefore, together with Kent Sikhs, we travelled to the Punjab in India and spent time in the very localities these Sikhs migrated from. We conducted fieldwork in their villages and in a range of mental health care settings in Punjab communities, and in the large mental health hospital in Amritsar.

Through two examples, we demonstrate the potential of ethnography to unearth or identify issues that would not be uncovered through other research methods or routine psychiatric assessments. One of the things that has come out very powerfully from our ethnographic work is the importance of intergenerational experiences; the migration journeys, literal or metaphorical, live on through subsequent generations – instantiated in social, psychological and biological phenotypes. While family, developmental and social histories are important in psychiatry, these have a far more nuanced relevance in ethnic minority patients. The understandings of mental health are reiterated across generations: Sikhs inherit perceptions from their parents who migrated to the UK, and Sikhs born in the UK pass on such interpretations to their children. Our ethnography clearly demonstrates the intergenerational transfer of thoughts, behaviours, customs and values of mental health – the transmission of certain cultural conventions and rituals, and the lack of transfer of other things which are much more related to the wider British culture. Jagvir, a 24-year-old study participant, summarised this aptly during our fieldwork:

‘No one ever really talks about their mental health problems. I think it is a flaw. Maybe ‘a flaw’ is the wrong word. It is just something that is not done. If your grandad does not speak about them, and then my dad, for example, my parents, I do not think I have ever seen them say that I was depressed during this period or even discussed it. Maybe they were, and they just did not discuss it with me. For me, you know, I have never seen people open up about mental health. Why would I then be inclined to open up about mental health? It is actually a generational thing. I think from my understanding of talking to my grandparents and my parents that they were the generations who had it much harder than me. I think me, growing up, I was in that lucky generation, I experienced some of it, maybe the name calling and being picked on individually, but I do not think I had the being spat on and being beaten up just for being different.’

A second unexpected finding was the high number of suicides in the Sikh community in which we conducted our fieldwork. This included a middle-aged man who set himself on fire in the parking lot of the local temple, a woman in her fifties who sent her husband to the grocery store and hanged herself with her scarf, and a teenage boy who jumped from a bridge on to a busy road. At least five Sikhs died by suicide in the first 6 months of our fieldwork. These suicides were not talked about during conventional interviews. It was because of the immersive nature of ethnographic fieldwork that we became aware of this. Indeed, during the first interviews many Sikhs stated that there were no problems with mental health in their community, or only offered some superficial insights. But, once the ethnographer gained the Sikhs’ trust, many talked about suicidal thoughts and experiences of suicide – a topic we were not planning to focus on when we started the study.

Importantly, when we sought to find data on suicide by ethnicity, a gap became clear. In the UK, there are currently no public health data on suicide by ethnicity as there is also no obligation to record ethnicity on death certificates.^5^ Most likely, there were more suicides in this specific Sikh community and this gap urgently needs to be addressed through the addition of this information on death certificates.

In conclusion, it is important to note that good ethnography is not just merely describing a community and their understandings of mental health. Much like psychiatry, it is in essence a critical examination of the data. This is reflected in the many concepts that anthropology introduced into health research and medical practice^6^ (for instance, structural violence and systemic racism).^7^ If we are to take the urgent calls for intersectional evidence seriously, then ethnographic data are part of the solution; surveys, clinical trials and mechanistic studies only offer part of the answer. Theory and methods from anthropology, in tandem with psychiatry, offer powerful tools to establish a robust evidence base of how to address mental health inequalities in ethnic minority communities. Indeed, perhaps psychiatry is ethnography.

## References

[1] Miner HM. Body ritual among the Nacirema. Am Anthropol 1956: 58: 503–7.

[2] Dikomitis L , Wenning B. Ethnography: sense and sensibilities. Starting Res Clin Educ 2023; 8:145–54.

[3] Dikomitis L. Cyprus and Its Places of Desire: Cultures of Displacement among Greek and Turkish Cypriot Refugees. Bloomsbury Publishing, 2012.

[4] Dikomitis L , Shergill S , Dikomitis E. Sikh Silences across Generations. Anthropology News, 12 Jan 2024 (https://www.anthropology-news.org/articles/sikh-silences-across-generations/).

[5] Cohen J , Katona C , Bhugra D. National data on suicide must include ethnicity. BMJ 2020: 27: 371. 10.1136/bmj.m4105 33109680

[6] Panter-Brick C , Eggerman M. The field of medical anthropology in Social Science & Medicine. Soc Sci Med 2018: 196: 233–9. 29137936 10.1016/j.socscimed.2017.10.033

[7] Farmer P. An anthropology of structural violence. Curr Anthropol 2004: 45: 305–25.

